# P10 The yeast of our worries: a challenging case of persistent candidaemia following emergency abdominal surgery

**DOI:** 10.1093/jacamr/dlad143.014

**Published:** 2024-01-03

**Authors:** Melanie Etti, Aarash Ahmadi, Clare Brandish, Karthiga Sithamparanathan, Nick Wong, Ruby Devi, Jean O’Driscoll

**Affiliations:** Department of Microbiology, Stoke Mandeville Hospital, Aylesbury, UK; Medical Sciences Division, University of Oxford, Oxford, UK; Pharmacy Department, Stoke Mandeville Hospital, Aylesbury, UK; Pharmacy Department, Stoke Mandeville Hospital, Aylesbury, UK; Department of Microbiology, Stoke Mandeville Hospital, Aylesbury, UK; Department of Microbiology, Stoke Mandeville Hospital, Aylesbury, UK; Department of Microbiology, Stoke Mandeville Hospital, Aylesbury, UK; Department of Microbiology, Stoke Mandeville Hospital, Aylesbury, UK

## Abstract

**Case summary:**

A 47-year-old woman with a past medical history of osteoarthritis only presented to hospital with abdominal pain three days following a diagnostic colonoscopy performed for suspected bowel malignancy. Upon admission, she was diagnosed with a bowel perforation and subsequently underwent an emergency laparotomy and anterior resection for a newly diagnosed sigmoid adenocarcinoma. Her recovery was complicated by an anastomotic leak requiring multiple washouts due to faeculent peritoneal soiling. Following surgery, she was admitted to the ICU with an open abdomen for vacuum-assisted wound closure and mesh-mediated fascial traction. A sample of peritoneal fluid taken during her initial washout grew *Escherichia coli*, for which she was initially treated with piperacillin/tazobactam, and later, meropenem, after another *E. coli* isolate from a sample of intra-abdominal pus collected during a subsequent washout demonstrated piperacillin/tazobactam resistance. Despite treatment with meropenem, the patient continued to have intermittent fevers up to 40°C. Blood cultures taken during a febrile episode grew yeasts identified as *Candida glabrata*, at which point anidulafungin was commenced empirically. Computed tomography imaging of the chest, abdomen and pelvis did not reveal any potential source of invasive candidiasis. To exclude line infection as the source of the *Candida*, all central lines were removed, leaving only peripheral venous access. Despite this, the candidaemia continued. In total, 18 blood cultures taken over a period of 19 days grew *C. glabrata*. After 7 days of empirical treatment with anidulafungin, voriconazole was added in view of the persistent elevation in the patient’s serum β-D-glucan (BDG) and recurrent isolation of *C. glabrata* from blood cultures. Flucytosine was added after a further 10 days with no effect on the patient’s clinical status. Following removal of the abdominal mesh and the eventual closure of the patient’s abdomen, the time to positivity for her blood cultures gradually increased and eventually became negative at 5 days. Flucytosine was then stopped and anidulafungin and voriconazole were continued. A sustained decline in the patient’s serum BDG was observed thereafter and antifungal treatment was eventually stopped after three negative BDG measurements were obtained.^1^

**Conclusions:**

Fungal mesh infection is a rare but serious complication of abdominal surgery involving mesh insertion.^2^ To our knowledge, this is the first reported case of abdominal surgical mesh infection caused by *C. glabrata*. Previously reported cases in the literature have been due to other *Candida* spp. (*albicans*, *krusei* and *norvegensis*), *Aspergillus* and *Coccidioides* spp.^1–3^  *C. glabrata* is particularly adept at biofilm formation, causing low therapeutic response to antifungal treatment.^4^ In this case, source control could not be readily achieved as the mesh was required to aid abdominal closure, resulting in treatment failure despite triple antifungal therapy. This case serves as a stark reminder of the devastating and potentially fatal consequences that may result from this surgical complication and the perils associated with the use of mesh in abdominal surgery when the peritoneum has been soiled.
